# Palliative gastrectomy is beneficial in selected cases of metastatic gastric cancer

**DOI:** 10.1186/s12904-017-0192-1

**Published:** 2017-03-14

**Authors:** Jun-Te Hsu, Jian-Ann Liao, Huei-Chieh Chuang, Tai-Di Chen, Tsung-Hsing Chen, Chia-Jung Kuo, Chun-Jung Lin, Wen-Chi Chou, Ta-Sen Yeh, Yi-Yin Jan

**Affiliations:** 1 Department of Surgery, Chang Gung Memorial Hospital at Linkou, Chang Gung University College of Medicine, #5, Fushing Street, Kweishan District, Taoyuan City, 333 Taiwan; 2 Department of Pathology, Chang Gung Memorial Hospital at Linkou, Chang Gung University College of Medicine, Taoyuan City, 333 Taiwan; 3 Department of Gastroenterology, Chang Gung Memorial Hospital at Linkou, Chang Gung University College of Medicine, Taoyuan City, 333 Taiwan; 4 Department of Hematology-Oncology, Chang Gung Memorial Hospital at Linkou, Chang Gung University College of Medicine, Taoyuan City, 333 Taiwan

**Keywords:** Metastatic gastric cancer, Palliative gastrectomy, Metastasectomy, Salvage chemotherapy, Survival

## Abstract

**Background:**

Salvage chemotherapy is the mainstay of treatment for metastatic gastric cancer (mGC). This study aimed to clarify the effects of palliative gastrectomy (PG) and identify prognostic factors in mGC patients undergoing PG.

**Methods:**

This was a retrospective review of 333 mGC patients receiving PG or a non-resection procedure (NR) between 2000 and 2010. Clinicopathological factors affecting the prognosis of these patients were collected prospectively and analyzed.

**Results:**

One hundred and ninety-three patients underwent PG and 140 NR. The clinicopathological characteristics were comparable between the two groups except for metastatic pattern. There were no significant differences in postoperative morbidity and mortality between the two groups. The PG group had a significantly longer median overall survival compared with the NR group (7.7 months vs. 4.9 months). In the PG group, age ≤58 years, preoperative albumin level >3 g/dL, ratio of metastatic to examined lymph nodes ≤0.58, and administration of chemotherapy were independent prognostic factors in multivariate analysis.

**Conclusions:**

Patients undergoing PG had better outcomes than those undergoing NR. Among the patients undergoing resection, age ≤58 years, a better preoperative nutritional status, less nodal involvement and postoperative chemotherapy independently affected patient survival.

## Background

Even though the incidence of gastric cancer (GC) has decreased over the past 3 decades, it was still the third leading cancer-related cause of death worldwide in 2012 [1]. Surgical resection with adequate lymphadenectomy provides the best chance of a cure [2, 3]. However, most patients present with locally advanced or metastatic disease at the time of diagnosis, with a 5-year survival rate of around 10% [4, 5]. Patients with inoperable or metastatic disease usually die within 12 months with or without salvage chemotherapy [6, 7]. However, patients with tumor-associated symptoms including dysphagia, gastric outlet obstruction, bleeding or gastric perforation may need a surgical intervention. Our previous studies showed that although GC tends to exhibit a more aggressive tumor behavior in young patients than in old patients, young patients with metastatic disease undergoing palliative gastrectomy (PG) have better outcomes than old patients [8]. A systemic review and meta-analysis of retrospective non-randomized studies indicated that PG may be beneficial compared with non-resection treatment for patients with metastatic GC (mGC); however, questions regarding which patients are suitable for PG remain unanswered [9]. Therefore, the aims of the present study were to detail the clinicopathological parameters that objectively affect clinicians’ decision-making, elucidate postoperative morbidity and mortality, and determine the prognostic factors for selecting appropriate candidates for PG in a tertiary medical center.

## Methods

### Patients and surgical procedures

Between 2000 and 2010, 333 pathologically proven mGC patients undergoing PG or a non-resection procedure (NR) in Taiwan were enrolled. In general, gastrectomy was not performed in the patients who did not have tumor-associated symptoms or in those with peritoneal metastasis for which macroscopic curative resection was not expected. The patients with tumor-related symptoms or solitary distant visceral organ metastasis such as the ovary or liver for which complete resection of the metastatic tumor was feasible underwent PG (D1 or D2 lymphadenectomy) or gastrectomy (D2 lymphadenectomy) plus metastasectomy. The NRs included bypass surgery, laparoscopic/laparotomy biopsy, feeding jejunostomy, hemostasis (suture ligation of a bleeder), and gastrorrhaphy (repair of a perforation). No patient received preoperative chemotherapy or stent placement for obstruction symptoms. The tumors were staged according to the seventh edition of the American Joint Committee on Cancer Tumor Node Metastasis classification [10]. Suitable patients received salvage chemotherapy with fluoropyrimidine-based or platinum-based regimens.

### Clinical data collection

Patient demographics, clinicopathological features, Charlson comorbidity index score and surgical outcomes were compared between the PG and NR groups. The median follow-up times in the PG and NR groups were 7.2 months and 4.7 months, respectively. The overall survival of the patients in the PG group was evaluated and compared with that of the NR patients operated on during the same time period. The patients who died during the same hospitalization after surgery were included in the survival analysis. Survival duration was calculated from the time of surgery to death or the last follow-up date (August 31, 2012).

### Statistical analysis

Non-binomially distributed data are presented as median (range). Clinical records were compared using the Student’s *t*-test or Pearson’s chi-square test, as appropriate. Patient GC-specific survival was estimated using the Kaplan-Meier method, and differences between subgroups were assessed using the log-rank test. Potentially important factors obtained using univariate analysis (*P* <0.1) were included in multivariate analysis, and both analyses were performed using a Cox proportional hazards model. A *P* value of less than 0.05 was considered to be statistically significant. All statistical analyses were performed using the Statistical Package for the Social Sciences version 20.0 for Windows (SPSS Inc., Chicago, IL, USA).

## Results

As shown in Table 1, no significant differences were found between the PG and NR groups in terms of age, gender, platelet count, hemoglobin, levels of albumin, alanine aminotransferase, aspartate aminotransferase, alkaline phosphatase, total bilirubin and creatinine, Charlson comorbidity index score, tumor-associated symptoms, and percentage of postoperative salvage chemotherapy. The PG group had a higher and lower proportion of hematogenous spread alone and peritoneal metastasis than the NR group, respectively (*P* <0.029). There were no differences in surgical complications and in-hospital mortality or mortality within 30 days after surgery between the two groups. The PG group had a higher percentage of patients who survived for more than 12 months compared with the NR group. (29.5% vs. 15.7%; *P* =0.005).

**Table 1:** Clinical data and outcomes of metastatic gastric cancer patients in terms of resectability

Parameters	Resection (*n* = 193)	Non-resection (*n* = 140)	*P* value
Age (years), median (range)	65 (26–89)	63 (22–91)	0.782
Gender			0.393
Male	126 (65.3)	85 (60.7)	
Female	67 (34.7)	55 (39.3)	
Platelet (10^3^/uL), median (range)	258.5 (58–680)	281 (73–622)	0.609
Hemoglobin (g/dL), median (range)	10.9 (3.8–20.6)	11.0 (4.0–16.5)	0.881
Albumin (g/dL), median (range)	3.7 (1.7–4.9)	3.7 (1.9–4.9)	0.135
AST (U/L), median (range)	18 (5–223)	19 (4–340)	0.491
ALT (U/L), median (range)	14 (1–156)	14 (2–106)	0.975
ALK-P (U/L), median (range)	67 (20–349)	64 (30–459)	0.998
Total bilirubin, (mg/dL) (range)	0.6 (0.2–1.8)	0.6 (0.2–2.0)	0.252
Creatinine (mg/dL), median (range)	0.9 (0.3–11.2)	1.0 (0.4–3.4)	0.500
Charlson comorbidity index score			0.529
2	89 (46.1)	74 (52.9)	
3	71 (36.8)	47 (33.6)	
4	21 (10.9)	10 (7.1)	
≥ 5	12 (6.2)	9 (6.4)	
Tumor-associated symptoms	110 (57.0)	82 (58.6)	0.774
Metastatic pattern			0.029
Hematogenous spread alone	56 (29.0)	26 (18.6)	
Peritoneum	137 (71.0)	114 (81.4)	
Complications	36 (18.7)	28 (20.0)	0.758
In-hospital mortality	20 (10.4)	13 (9.3)	0.745
Mortality within 1 month	14 (7.3)	6 (4.3)	0.260
Chemotherapy	124 (64.2)	83 (59.3)	0.357
Survival time (months)			0.005
≤ 6	82 (42.5)	81 (57.9)	
6 ~ 12	54 (28.0)	37 (26.4)	
> 12	57 (29.5)	22 (15.7)	

Figures are numbers with percentages in parentheses, unless otherwise stated

Hematogenous spread alone indicates metastases to the distant organ or distant nodes

Tumor-associated symptoms include dysphagia, obstruction, bleeding or perforation

*ALT* alanine aminotransferase, *AST* aspartate aminotransferase, *ALK-P* alkaline phosphatase

**Fig. 1:**
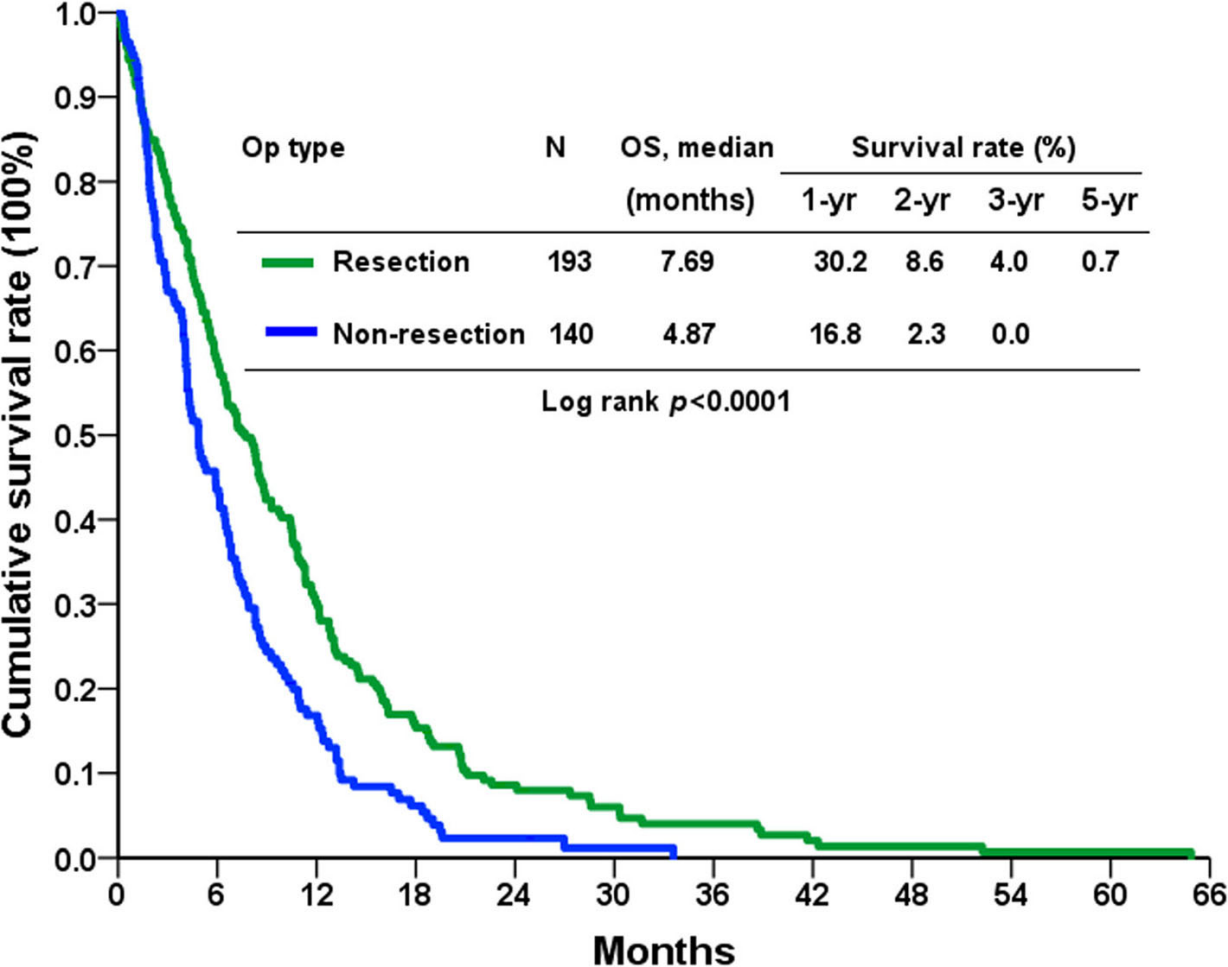
Overall survival (OS) rates of patients with metastatic gastric cancer in terms of resectability

**Fig. 2:**
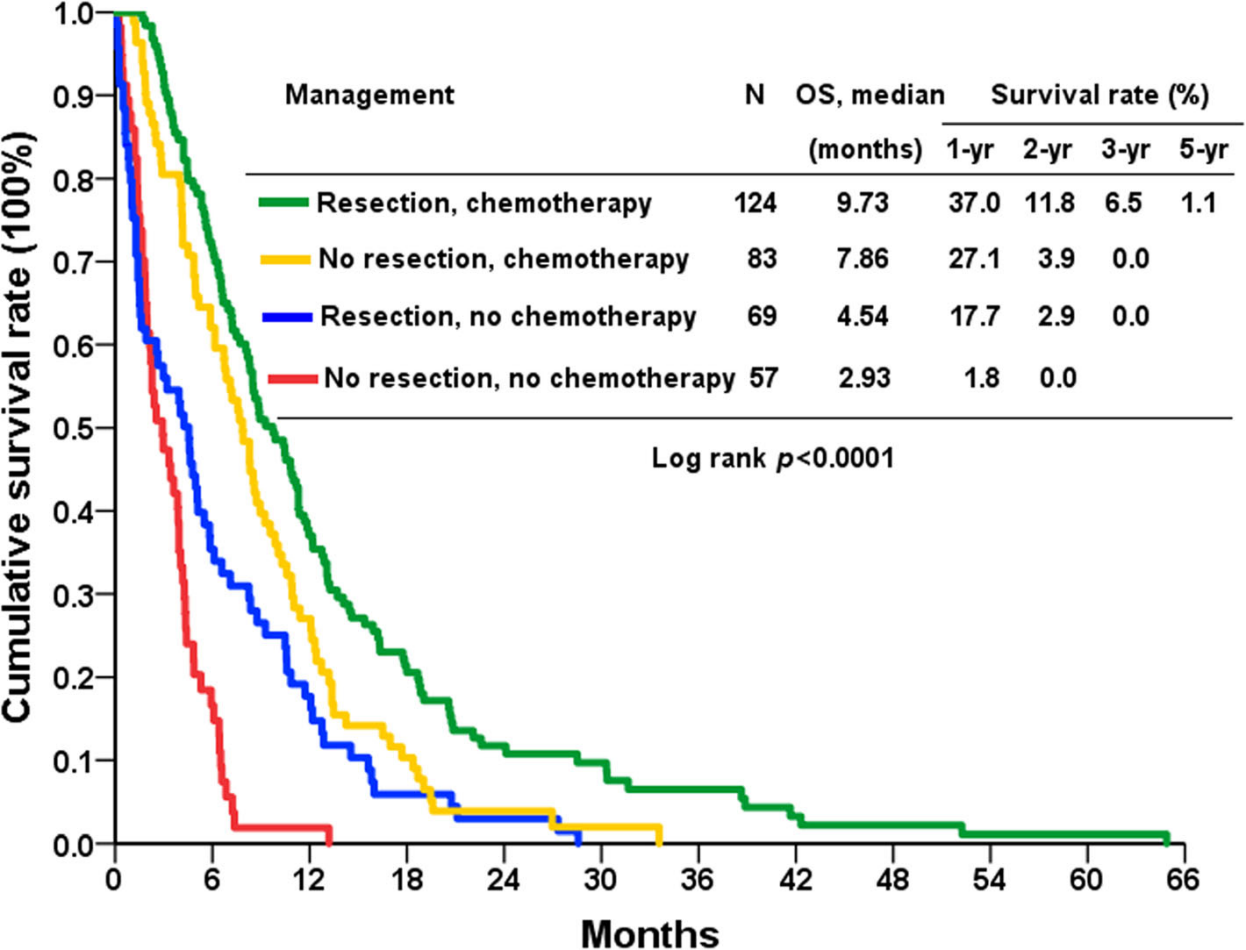
Overall survival (OS) rates of patients with metastatic gastric cancer in terms of resection and chemotherapy

The patients in the PG group had a significantly longer median overall survival time compared with those in the NR group (7.7 months vs. 4.9 months; *P* <0.0001; Fig. 1). The overall survival rates in the PG group at 1, 2, and 3 years were 30.2, 8.6, and 4.0%, respectively. The patients treated with PG and postoperative salvage chemotherapy had a markedly longer median overall survival time than those receiving PG or salvage chemotherapy alone, or the NR group without chemotherapy (*P* <0.0001; Fig. 2). The 1-, 2-, and 3-year survival rates were 37.0, 11.8, and 6.5%, respectively, for patients undergoing PG and chemotherapy, and 2.9, 1.8 and 0% for patients without resection and chemotherapy. Thirteen and 6 patients underwent ovariectomy and hepatectomy in addition to gastrectomy (D2 lymphadenectomy), respectively. No mortality was noted in these patients. Among 17 patients with ovarian metastasis, thirteen patients undergoing PG along with resection of ovary had significantly longer median overall survival compared with 4 patients not undergoing resection (15.9 months vs. 5.9 months; *P* =0.027). The 1-, 3-, and 5-year survival rates were 61.5, 15.4, and 7.7%, respectively, in the patients who underwent ovariectomy, and the 1-, 2-, and 3-year survival rates were 66.7, 33.3, and 16.7%, respectively, in the patients who underwent hepatectomy.

Table 2 shows the univariate analysis of various clinicopathological factors associated with surgical outcomes in the PG group. Age, gender, albumin level, tumor-associated symptoms, nodal involvement, ratio of metastatic to examined lymph nodes, lymphatic and perineural invasion as well as the administration of postoperative chemotherapy significantly affected the prognosis. No significant differences in overall survival time were observed for levels of alkaline phosphatase and total bilirubin, the type of gastrectomy, tumor location, differentiation, Lauren’s histological type, depth of tumor invasion, number of lymph nodes retrieved, vascular invasion and metastatic pattern. In multivariate analysis, the independent prognostic predictors were age ≤58 years, a preoperative albumin level >3 g/dL, a ratio of metastatic to examined lymph nodes ≤0.58 and administration of salvage chemotherapy (Table 3).

**Table 2:** Univariate analysis of prognostic factors of metastatic gastric cancer patients undergoing resection

Factors	Median survival (months)	95% CI of median	1-year (%)	3-year (%)	*P* value
Age (years)					0.003
≤ 58 (*n* = 71)	10.85	9.03–12.67	42.3	5.1	
> 58 (*n* = 122)	6.15	5.28–7.02	23.2	3.4	
Gender					0.009
Male (*n* = 126)	7.23	5.42–9.05	23.8	1.3	
Female (*n* = 67)	10.98	6.19–15.77	41.8	8.3	
Albumin (g/dL)					0.001
≤ 3 (*n* = 37)	4.44	3.65–5.23	17.5	0.0	
> 3 (*n* = 129)	8.52	6.54–10.49	36.0	3.8	
ALK-P (U/L)					0.185
≤ 60 (*n* = 57)	10.82	8.63–13.01	42.1	4.4	
> 60 (*n* = 92)	7.10	5.33–8.87	26.1	3.3	
Total bilirubin (mg/dL)					0.359
≤ 1.3 (*n* = 153)	8.25	6.90–9.60	32.2	4.4	
> 1.3 (*n* = 6)	3.58	0.23–2.94	16.7	0.0	
Gastrectomy					0.525
total (*n* = 84)	8.52	5.39–11.64	33.4	3.5	
subtotal (*n* = 89)	6.61	5.05–8.17	27.4	4.3	
Location					0.161
upper (*n* = 44)	10.55	7.39–13.72	41.9	7.7	
middle (*n* = 29)	8.06	4.92–11.19	36.3	0.0	
lower (*n* = 97)	6.61	5.54–7.68	24.1	5.9	
diffuse (*n* = 22)	6.38	0.60–12.16	27.3	0.0	
Tumor-associated symptoms					0.008
no (*n* = 83)	12.63	10.04–15.23	40.4	4.3	
yes (*n* = 110)	8.88	7.21–10.56	22.7	4.1	
Differentiation					0.549
yes (*n* = 44)	8.52	5.99–11.04	34.1	2.3	
no (*n* = 149)	7.10	5.35–8.85	29.0	4.6	
Lauren’s classification					0.445
intestinal (*n* = 57)	8.91	6.44–11.38	31.6	4.0	
diffuse (*n* = 101)	6.94	4.99–8.88	27.2	3.8	
mixed (*n* = 31)	7.23	3.55–10.92	37.2	6.8	
T status					0.143
1/2/3 (*n* = 19)	10.85	8.08–13.62	36.8	15.8	
4 (*n* = 174)	7.17	5.63–8.701	29.4	3.4	
N status					0.042
0 (*n* = 12)	11.67	5.14–18.20	50.0	16.7	
1 (*n* = 12)	13.71	7.24–20.18	66.7	0.0	
2 (*n* = 32)	6.54	3.78–9.30	16.1	3.2	
3 (*n* = 137)	3.94	5.24–8.63	28.4	3.4	
LN ratio^a^					0.003
≤ 0.58 (*n* = 97)	10.39	8.12–12.66	35.4	5.9	
> 0.58 (*n* = 96)	5.56	4.24–6.88	28.9	2.2	
No. of lymph node retrieved					0.881
< =15 (*n* = 47)	5.03	2.47–7.59	27.7	6.4	
> 15 (*n* = 146)	8.32	6.94–9.70	30.9	3.1	
Resection margins					0.675
Positive (*n* = 52)	6.61	3.40–9.82	32.6	0.0	
Negative (*n* = 141)	8.06	6.53–9.58	29.3	4.8	
Vascular invasion					0.611
Positive (*n* = 70)	8.52	6.42–10.09	28.2	4.1	
Negative (*n* = 118)	7.17	5.28–9.05	27.8	3.1	
Lymphatic invasion					0.066
Positive (*n* = 171)	7.17	5.55–8.77	28.2	4.1	
Negative (*n* = 19)	11.67	7.56–15.79	47.4	5.3	
Perineural invasion					0.047
Positive (*n* = 147)	7.17	5.53–8.80	27.0	3.2	
Negative (*n* = 42)	9.27	4.78–13.77	41.8	7.4	
Metastatic pattern					0.891
Hematogenous spread alone (*n* = 56)	5.59	4.67–6.51	28.6	6.0	
Peritoneum (*n* = 137)	8.48	7.00–9.96	30.8	3.2	
Chemotherapy					<0.0001
No (*n* = 69)	4.54	2.72–6.36	17.7	0.0	
Yes (*n* = 124)	9.73	7.77–11.69	37.0	6.5	

Hematogenous spread alone indicates metastases to the distant organ or distant nodes

Tumor-associated symptoms include dysphagia, obstruction, bleeding or perforation

*ALK-P* alkaline phosphatase, *CI* confidence interval

^a^ratio of metastatic to examined lymph nodes

**Table 3:** Multivariate analysis of prognostic factors in metastatic gastric cancer patients undergoing resection

Factors	Hazard ratio (95% CI)	*P* value
Age (years)		
> 58/≤58	1.47 (1.01–2.13)	0.045
Gender		
Male/female	1.14 (0.80–1.63)	0.465
Albumin (g/dL)		
≤ 3 />3	1.93 (1.24–3.00)	0.003
Tumor-associated symptoms		
Yes/no	1.04 (0.73–1.47)	0.837
N status		
N1/N0	0.83 (0.33–2.10)	0.695
N2/N0	1.30 (0.56–3.01)	0.542
N3/N0	1.16 (0.49–2.72)	0.740
LN ratio^a^		
> 0.58/≤0.58	1.47 (1.01–2.15)	0.047
Lymphatic invasion		
Yes/no	1.20 (0.62–2.32)	0.588
Perineural invasion		
Yes/no	1.08 (0.70–1.65)	0.728
Chemotherapy		
No/yes	1.68 (1.19–2.38)	0.004

*CI* confidence interval

Tumor-associated symptoms include dysphagia, obstruction, bleeding or perforation

^a^ratio of metastatic to examined lymph nodes

## Discussion

In the present study, there were no differences in surgical complications and mortality rates between the PG and NR groups. The median overall survival time was longer in the mGC patients undergoing PG compared with NR. The patients receiving PG and postoperative salvage chemotherapy had better outcomes than those with other management. In the PG group, age ≤58 years, preoperative albumin level >3 g/dL, ratio of metastatic to examined lymph nodes ≤0.58 and administration of chemotherapy were independent prognostic factors.

It has been reported that young patients undergoing curative resection have a longer survival duration than old patients in multivariate analysis [11]. Our previous research also indicated that for patients with mGC, the younger patients (age ≤40 years) had significantly better outcomes than the older (age between 56 and 75 years) patients after PG [8]. Dittmr et al. reported that comparing mGC patients receiving PG with those receiving NR, an age <50 years was a predictor for improved survival in univariate analysis but not in multivariate analysis [12]. Lim et al. found that an age <60 years was associated with prolonged survival in mGC patients undergoing resection in univariate analysis [13]. Since the median age of patients in the PG group surviving more than 12 months was 58 years in the present study (data not shown), we selected the cutoff value 58 years as one of prognostic covariates. Our results demonstrate that an age ≤58 years was as a prognostic factor for mGC patients undergoing PG in multivariate analysis. We speculated that young patients who had better visceral organ functional reserve and less concomitant comorbidities than the old after PG than did in the old might therefore in part explain favorable outcomes in the young.

Koo et al. developed a prognostic model using 2805 patients with metastatic or recurrent GC undergoing chemotherapy, and found that Eastern Cooperative Oncology Group (ECOG) performance status ≥2, no gastrectomy, presence of peritoneal, bone, or lung metastases, high levels of serum alkaline phosphatase (>120 U/L) and total bilirubin (>1.2 mg/dL), and a low serum albumin level (<3.3 g/dL) were poor prognostic factors [14]. Lee et al. also reported an estimated median survival of <3 months in mGC patients receiving chemotherapy with more than four unfavorable factors (no gastrectomy, albumin <3.6 g/dL, alkaline phosphatase >85 U/L, ECOG performance status ≥2, presence of bone metastasis, ascites) [15]. The present study showed that apart from an age ≤58 years, a serum albumin level >3 g/dL, ratio of metastatic to examined lymph nodes ≤0.58, and administration of postoperative chemotherapy were independent predictors for survival in the mGC patients undergoing PG suggesting that patients with above-mentioned favorable factors may benefit greatly from PG.

It has been reported that a subgroup of GC patients with ovarian metastasis (Krukenberg tumors) may benefit from resection of the ovary when the gross tumors are thoroughly removed [16, 17]. Peng et al. reported that ovarian metastasectomy significantly prolonged the median overall survival in select GC patients without ascites (21 months vs. 13 months, *P*  =  0.008) or patients undergoing gastrectomy (19 months vs. 9 months, *P*  =  0.048) [16]. Ayhan et al. also found that survival was significantly superior in GC patients with ovarian metastasis who underwent cytoreduction.^17^ In the present study, thirteen patients with Krukenberg tumors alone underwent gastrectomy with D2 lymphadenectomy and ovariectomy, and the median survival time was 15.9 months (range 4.4 months to 64.9 months). Our results supported that PG along with resection of ovarian metastasis can improve patient’s overall survival compared with no ovariectomy (median, 15.9 vs. 5.9 months).

The management of GC patients with liver metastasis remains controversial. Although evidence supporting the role of hepatectomy in the treatment of these patients is weak, a survival advantage has been reported in a select group [18]. However, only 10–20% of GC patients with liver metastasis are candidates for hepatic resection [19]. The beneficial effects of hepatic resection or radiofrequency ablation for GC patients with synchronous liver metastasis have also been reported [20, 21], with 1-, 2-, and 3-year overall survival rates after resection of 70, 11, and 5%, respectively [20]. Furthermore, Cheon et al. also suggested that hepatic resection should be considered as an option for GC patients with liver metastasis [22]. The number of liver metastases has been shown to be an independent prognostic factor for patients after hepatectomy [21], and those with a solitary liver metastasis have been reported to have a better survival rate than those with multiple liver metastases [18, 21]. Recently, Tiberio et al. reported 1-, 3-, and 5-year survival rates of 50.4, 14.0, and 9.3%, respectively, for 53 GC patients with synchronous liver metastases who underwent gastrectomy and R0 hepatectomy, and 6.8, 2.3 and 0% for 44 patients after palliative surgery without resection [23]. Similar to these results, six patients in the current study with a solitary synchronous liver metastasis who underwent gastrectomy with D2 lymphadenectomy and R0 hepatectomy had a median survival of 20.8 months (range 6.6 months to 38.6 months) and 1-, 2-, and 3-year overall survival rates of 66.7, 33.3, and 16.7%, respectively.

For patients with metastatic disease, salvage chemotherapy is the mainstay of treatment, with reported median survival times ranging from 5.7 to 11.2 months, regardless of the chemotherapy regimen [24–26]. However, some patients with tumor-related complications, such as gastrointestinal obstruction, bleeding, and gastric perforation may necessitate surgery [27]. Our findings indicated that PG followed by postoperative salvage chemotherapy significantly prolonged the overall survival of the mGC patients compared to those without gastrectomy and chemotherapy. The median overall survival time of the patients with PG and chemotherapy was 9.7 months compared to 4.5 months for those who received PG alone. Of note, the patients in the NR group did not receive chemotherapy; the median overall survival time was only 2.9 months. Therefore, we suggest that in highly select patients with metastatic disease, PG and chemotherapy should be considered not only to relieve tumor-associated symptoms and improve quality of life but also to enhance survival benefit. This is also supported by other studies [9, 23, 28–30]. The possible reason why PG improves patient outcomes might be associated with reducing tumor burden and rendering the patients more responsive to salvage chemotherapy. In addition, it has been shown that cytoreductive surgery diminishes a hypercatabolic state and confers immunological benefits through decreasing the release of tumor-derived immunosuppressive cytokines [31]. However, further research is needed to prove this hypothesis because selection bias/confounder exists in the current study.

The limitations of the present study are its retrospective design and that patients were enrolled in a single institution. The PG group had a significantly higher proportion of hematogenous spread alone and lower proportion of peritoneal metastasis than the NR group (*P* =0.029). Of note, compared with the NR group, the PG group did not increase surgical mortality and had longer survival. However, Tokunaga et al. reported that patients with peritoneal metastasis did not benefit from PG [32, 33]. To confirm whether PG can provide survival benefits in patients with mGC, two large randomized controlled clinical studies are performed [34, 35]. Fujitani K et al. indicated that gastrectomy (restricted to D1 lymphadenectomy without any resection of metastatic lesions, R2 resection) followed by salvage chemotherapy did not provide survival benefit compared with chemotherapy alone in mGC with a single non-curable factor (confined to the liver, peritoneum, or para-aortic lymph nodes) [34]. Different from their studies, we performed gastrectomy with D2 lymphadenectomy and metastasectomy to achieve grossly R0 resection in some patients with synchronous solitary metastasis. In addition, select patients with tumor-associated symptoms including dysphagia, gastric outlet obstruction, bleeding or perforation were treated by PG to relieve symptoms. Our results also indicated that mGC patients receiving PG and salvage chemotherapy had better survival than salvage chemotherapy alone. Therefore, based on our current results, we suggest that PG should be considered in patients with favorable factors when only solitary metastasis was detected.

## Conclusions

Among the mGC patients undergoing palliative resection, age ≤58 years, a better pre-operative nutritional status, less nodal involvement and administration of chemotherapy were independent prognostic factors in multivariate analysis. The patients treated with a combination of PG and salvage chemotherapy had a longer survival time than those who received other management strategies. We recommend that mGC patients with these favorable prognostic factors and favorable general performance status should undergo PG with R0 metastasectomy if achievable followed by salvage chemotherapy.

## Abbreviations

ECOG: Eastern Cooperative Oncology Group
mGC: Metastatic gastric cancer
NR: Non-resection procedure
PG: Palliative gastrectomy

## References

[1] Ferlay J, Soerjomataram I, Dikshit R, Eser S, Mathers C, Rebelo M (2015). Cancer incidence and mortality worldwide: sources, methods and major patterns in GLOBOCAN 2012. Int J Cancer.

[2] Hsu JT, Lin CJ, Sung CM, Yeh HC, Chen TH, Chen TC (2013). Prognostic significance of the number of examined lymph nodes in node-negative gastric adenocarcinoma. Eur J Surg Oncol.

[3] Cheng CT, Tsai CY, Hsu JT, Vinayak R, Liu KH, Yeh CN (2011). Aggressive surgical approach for patients with T4 gastric carcinoma: promise or myth?. Ann Surg Oncol.

[4] Wang F, Chang YC, Chen TH, Hsu JT, Kuo CJ, Lin CJ (2014). Prognostic significance of splenectomy for gastric cancer patients undergoing total gastrectomy: a retrospective cohort study. Int J Surg.

[5] Tegels JJ, De Maat MF, Hulsewé KW, Hoofwijk AG, Stoot JH (2014). Improving the outcomes in gastric cancer surgery. World J Gastroenterol.

[6] Hsu JT, Chen TC, Tseng JH, Chiu CT, Liu KH, Yeh CN (2011). Impact of HER-2 overexpression/amplification on the prognosis of gastric cancer patients undergoing resection: a single-center study of 1,036 patients. Oncologist.

[7] Takeno A, Takiguchi S, Fujita J, Tamura S, Imamura H, Fujitani K (2013). Clinical outcome and indications for palliative gastrojejunostomy in unresectable advanced gastric cancer: multi-institutional retrospective analysis. Ann Surg Oncol.

[8] Hsieh FJ, Wang YC, Hsu JT, Liu KH, Yeh CN, Yeh TS (2012). Clinicopathological features and prognostic factors of gastric cancer patients aged 40 years or younger. J Surg Oncol.

[9] Lasithiotakis K, Antoniou SA, Antoniou GA, Kaklamanos I, Zoras O (2014). Gastrectomy for stage IV gastric cancer. a systematic review and meta-analysis. Anticancer Res.

[10] Edge SB, Byrd DR, Compton CC, Fritz AG, Greene FL, Trotti A, American Joint Committee on Cancer (2009). AJCC Cancer Staging Manual.

[11] Park JC, Lee YC, Kim JH, Kim YJ, Lee SK, Hyung WJ (2009). Clinicopathological aspects and prognostic value with respect to age: an analysis of 3,362 consecutive gastric cancer patients. J Surg Oncol.

[12] Dittmar Y, Rauchfuss F, Goetz M, Jandt K, Scheuerlein H, Heise M (2012). Non-curative gastric resection for patients with stage 4 gastric cancer--a single center experience and current review of literature. Langenbecks Arch Surg.

[13] Lim S, Muhs BE, Marcus SG, Newman E, Berman RS, Hiotis SP (2007). Results following resection for stage IV gastric cancer; are better outcomes observed in selected patient subgroups?. J Surg Oncol.

[14] Koo DH, Ryoo BY, Kim HJ, Ryu MH, Lee SS, Moon JH (2011). A prognostic model in patients who receive chemotherapy for metastatic or recurrent gastric cancer: validation and comparison with previous models. Cancer Chemother Pharmacol.

[15] Lee J, Lim T, Uhm JE, Park KW, Park SH, Lee SC (2007). Prognostic model to predict survival following first-line chemotherapy in patients with metastatic gastric adenocarcinoma. Ann Oncol.

[16] Peng W, Hua RX, Jiang R, Ren C, Jia YN, Li J (2013). Surgical treatment for patients with Krukenberg tumor of stomach origin: clinical outcome and prognostic factors analysis. PLoS One.

[17] Ayhan A, Guvenal T, Salman MC, Ozyuncu O, Sakinci M, Basaran M (2005). The role of cytoreductive surgery in nongenital cancers metastatic to the ovaries. Gynecol Oncol.

[18] Grimes N, Devlin J, Dunne DF, Poston G, Fenwick S, Malik H (2014). The role of hepatectomy in the management of metastatic gastric adenocarcinoma: a systematic review. Surg Oncol.

[19] Okano K, Maeba T, Ishimura K, Karasawa Y, Goda F, Wakabayashi H (2002). Hepatic resection for metastatic tumors from gastric cancer. Ann Surg.

[20] Chen J, Tang Z, Dong X, Gao S, Fang H, Wu D (2013). Radiofrequency ablation for liver metastasis from gastric cancer. Eur J Surg Oncol.

[21] Qiu JL, Deng MG, Li W, Zou RH, Li BK, Zheng Y (2013). Hepatic resection for synchronous hepatic metastasis from gastric cancer. Eur J Surg Oncol.

[22] Cheon SH, Rha SY, Jeung HC, Im CK, Kim SH, Kim HR (2008). Survival benefit of combined curative resection of the stomach (D2 resection) and liver in gastric cancer patients with liver metastases. Ann Oncol.

[23] Tiberio GA, Baiocchi GL, Morgagni P, Marrelli D, Marchet A, Cipollari C (2015). Gastric cancer and synchronous hepatic metastases: is it possible to recognize candidates to R0 resection?. Ann Surg Oncol.

[24] Cunningham D, Starling N, Rao S, Iveson T, Nicolson M, Coxon F (2008). Capecitabine and oxaliplatin for advanced esophagogastric cancer. N Engl J Med.

[25] Wagner AD, Grothe W, Haerting J, Haerting J, Kleber G, Grothey A (2006). Chemotherapy in advanced gastric cancer: a systematic review and meta-analysis based on aggregate data. J Clin Oncol.

[26] Kanat O, O’Neil BH (2013). Metastatic gastric cancer treatment: a little slow but worthy progress. Med Oncol.

[27] Cunningham SC, Schulick RD (2007). Palliative management of gastric cancer. Surg Oncol.

[28] Sun J, Song Y, Wang Z, Chen X, Chen X, Gao P, Xu Y (2013). Clinical significance of palliative gastrectomy on the survival of patients with incurable advanced gastric cancer: a systemic review and meta-analysis. BMC Cancer.

[29] Sougoultzis S, Syrios J, Xynos ID, Bovaretos N, Kosmas C, Sarantonis J (2011). Palliative gastrectomy and other factors affecting overall survival in stage IV gastric adenocarcinoma patients receiving chemotherapy: a retrospective analysis. Eur J Surg Oncol.

[30] Chen S, Li YF, Feng XY, Zhou ZW, Yuan XH, Chen YB (2012). Significance of palliative gastrectomy for late-stage gastric cancer patients. J Surg Oncol.

[31] Pollock RE, Roth JA (1989). Cancer-induced immunosuppression: implications for therapy?. Semin Surg Oncol.

[32] Tokunaga M, Terashima M, Tanizawa Y, Bando E, Kawamura T, Yasui H (2012). Survival benefit of palliative gastrectomy in gastric cancer patients with peritoneal metastasis. World J Surg.

[33] Kim KW, Chow O, Parikh K, Blank S, Jibara G, Kadri H (2014). Peritoneal carcinomatosis in patients with gastric cancer, and the role for surgical resection, cytoreductive surgery, and hyperthermic intraperitoneal chemotherapy. Am J Surg.

[34] Fujitani K, Yang HK, Mizusawa J, Kim YW, Terashima M, Han SU (2016). Gastrectomy plus chemotherapy versus chemotherapy alone for advanced gastric cancer with a single non-curable factor (REGATTA): a phase 3, randomised controlled trial. Lancet Oncol.

[35] Kerkar SP, Kemp CD, Duffy A, Kammula US, Schrump DS, Kwong KF (2009). The GYMSSA trial: a prospective randomized trial comparing gastrectomy, metastasectomy plus systemic therapy versus systemic therapy alone. Trials.

